# Pre-exposure prophylaxis against HIV: pros and cons

**DOI:** 10.1186/1742-4690-9-S1-I6

**Published:** 2012-05-25

**Authors:** Mark Wainberg

**Affiliations:** 1 McGill University, Montreal, Canada

## 

The successful use of the co-formulation of Tenofovir (TDF) and Emtricitabine (FTC) to prevent HIV infection among gay men was successfully demonstrated in 2010 in the iPREX trial. Moreover, this randomized placebo-controlled study showed that men who were adherent in regard to the once-daily use of TDF/FTC were at low risk for acquisition of HIV in comparison to both non-treated controls and men who were not as adherent to their recommended drug intake (44% protection overall but 92% protection among adherent populations). Similar findings were obtained in a different study termed Partners/Prep performed in two developing countries. Yet, despite these positive results, the idea of PrEP remains controversial in some settings, and the following concerns among others have been expressed.

1. The use of antiretroviral drugs in PrEP may deprive some people already infected by HIV of the chance to benefit from these same drugs, due to competition for resources and/or drug supply.

2. The use of PrEP will promote sexual promiscuity and lead to more STDs.

3. Although PrEP has worked in controlled trials, it will be less likely to be successful in real world settings, where adherence and counselling may be less than adequate,

4. PrEP may promote HIV drug resistance in real world settings if ARVs are taken by people who may already be HIV-infected and don´t know it.

5. Not all PrEP trials have been successful, e.g. VOICE and FEM-PrEP.

6. The daily consumption of ARVs may be toxic.

One potential solution to some of these concerns may be the use of PrEP on an intermittent basis, in which ARVs are taken only on the day of anticipated sexual relations and for two days after a sexual encounter. This concept could have the advantage of lowering both costs and drug-related toxicities.

